# Sex Differences in Strength, Self-Estimation, and Pain Perception Based on Physical Activity After Rotator Cuff Repair

**DOI:** 10.3390/healthcare13131624

**Published:** 2025-07-07

**Authors:** Zebin Wen, Yonghwan Kim, Yongchul Choi, Moonyoung Choi

**Affiliations:** 1 College of Physical Education, Taiyuan University of Technology, Taiyuan 030024, China; wenzebin@tyut.edu.cn; 2 Department of Physical Education, Gangneung-Wonju National University, Gangneung 25457, Republic of Korea; yhkim@gwnu.ac.kr; 3 Laboratory of Integrated Physiology, Department of Health and Human Performance, University of Houston, Houston, TX 77204, USA; 4 Department of Physical Education, Daegu University, Gyeongsan 38453, Republic of Korea

**Keywords:** rotator cuff, gender, physical activity, recovery, pain, rehabilitation

## Abstract

**Background:** The role of physical activity in early recovery following arthroscopic rotator cuff repair (ARCR) remains unclear, particularly regarding potential sex differences. This study examined the effects of physical activity on pain, self-estimation, and strength recovery after ARCR in middle-aged and older adults. **Methods:** Patients who underwent ARCR were classified into high physical activity (HPA, n = 94) and low physical activity (LPA, n = 99) groups based on the International Physical Activity Questionnaire. The Visual Analog Scale (VAS) for pain and the American Shoulder and Elbow Surgeons (ASESs) score for self-estimation were assessed preoperatively and at 6, 12, and 24 weeks postoperatively. Isokinetic shoulder strength was measured at the same intervals except for the 6-week assessment. **Results:** All groups demonstrated significant improvements in pain reduction and self-estimation over time (*p* < 0.05). At 6 and 12 weeks, the HPA group showed significantly lower VAS scores and higher ASES scores than the LPA group (*p* < 0.05). Notably, at 12 weeks, men in the LPA group exhibited pain levels comparable to the HPA group, whereas women in the LPA group continued to report significantly higher pain levels (*p* < 0.05). Isokinetic strength assessment revealed greater flexion and external rotation strength in the HPA group at 12 weeks (*p* < 0.05), though no significant between-group differences were observed at 24 weeks. **Conclusions:** Higher physical activity levels were associated with better early pain relief and self-estimation after ARCR, particularly within the first 12 weeks. These findings suggest that structured physical activity may enhance postoperative outcomes, with potential sex-based differences in pain perception.

## 1. Introduction

**Figure 1:**
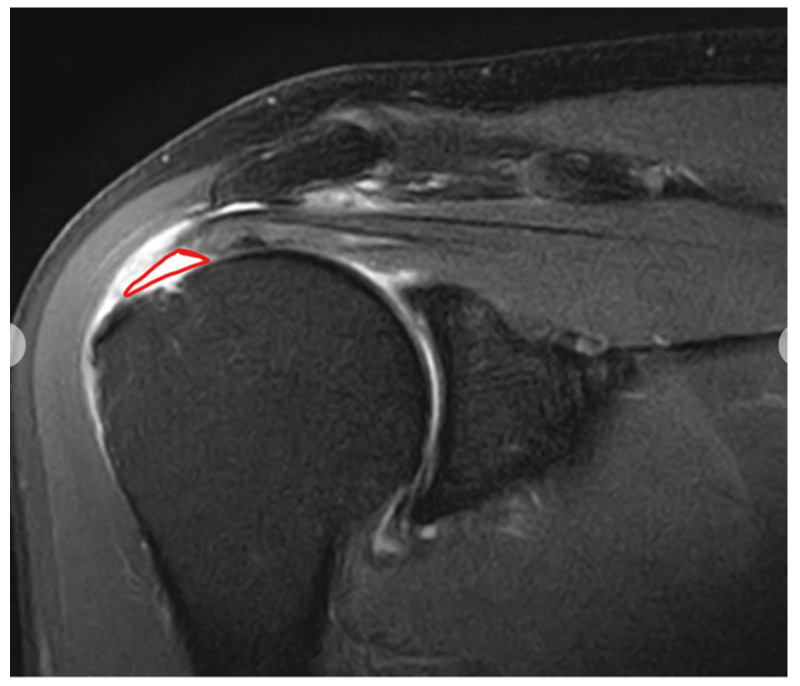
Rotator cuff tear image.

Rotator cuff tears are one of the leading causes of shoulder dysfunction, resulting in severe pain, muscle weakness, and significant limitations in activities of daily living (Figure 1) [1]. The prevalence of rotator cuff tears ranges from 6.8% to 22.4% in individuals over 40 years old, and it increases with age [2]. Consequently, they are recognized as a major clinical concern, particularly among middle-aged and older adults.

When conservative treatments such as rehabilitation therapy, pharmacologic management, and injections fail to provide sufficient pain relief and functional recovery, arthroscopic rotator cuff repair (ARCR) is recommended [3]. ARCR is widely performed as a minimally invasive approach, offering advantages such as reduced surgical trauma, faster recovery, and early restoration of shoulder range of motion [4]. However, patients often experience significant postoperative pain during the early recovery phase, leading to decreased physical activity levels and potential delays in functional recovery [5].

Postoperative pain management is a crucial factor influencing functional recovery and rehabilitation. Traditionally, oral opioid analgesics have been prescribed for pain control following ARCR [6]. However, prolonged opioid use is associated with various adverse effects, including nausea, vomiting, bowel obstruction, and urinary retention. In severe cases, opioid-related complications such as respiratory depression, confusion, and dizziness may further hinder the recovery process [7]. Given these risks, alternative strategies for effective pain management that minimize opioid use are needed.

Non-opioid-based approaches, including nonsteroidal anti-inflammatory drugs (NSAIDs) and intra-articular local anesthesia, have been proposed as viable alternatives [8,9]. However, these methods also have potential drawbacks, such as impairing tissue healing and bone metabolism, necessitating cautious use. Therefore, there is increasing interest in identifying safe and effective non-pharmacological strategies for pain control and early postoperative recovery.

Among various pain management approaches, physical activity has been extensively studied and recognized as an effective intervention for pain reduction [10,11]. Previous studies have demonstrated that physical activity induces a hypoalgesic response, termed exercise-induced hypoalgesia (EIH), which reduces pain sensitivity both during and after exercise [12]. EIH is mediated by the activation of descending inhibitory pain pathways and the release of endogenous opioids, along with an increase in anti-inflammatory cytokines such as interleukin-10 (IL-10) [13,14]. These mechanisms suggest that maintaining an adequate level of physical activity may facilitate pain modulation and recovery after ARCR.

However, during the early postoperative period, patients are required to immobilize the shoulder using an abduction brace for 4 to 6 weeks to protect the repaired tendon and minimize tension at the repair site [15]. Immobilization has been reported to promote vascularization and facilitate tendon healing, which is why it is widely recommended following ARCR [16]. Despite these benefits, prolonged immobilization restricts upper limb movement, limiting patients’ ability to perform daily activities. This reduction in physical activity may contribute to delayed functional recovery and hinder the early return to daily life.

Previous studies on clinical outcomes following ARCR have primarily focused on pain management, the restoration of shoulder and upper extremity function, and the successful healing of the repaired tendon [17,18]. Existing research has largely examined factors such as tear size, patient-related characteristics, and surgical techniques to predict these outcomes [18]. However, studies systematically analyzing the impact of physical activity levels on postoperative recovery remain limited, particularly with respect to sex-based differences. Daniels et al. reported that female patients experienced higher levels of pain and exhibited a relatively delayed functional recovery during the first three months after ARCR compared to male patients [19]. Rice et al. also reviewed sex differences in EIH, highlighting that while females tend to report greater pain sensitivity in some contexts, current evidence regarding sex-specific differences in EIH remains inconclusive [20]. These mixed findings suggest that sex-related variations in pain modulation through physical activity are complex and not yet clearly understood. We therefore treated sex not merely as a confounder but as an a priori variable of interest, aiming to elucidate its moderating role in the relationship between physical activity and early postoperative outcomes. This rationale informed our analytical approach and underscores the value of incorporating sex-based comparisons in ARCR recovery research.

The present study aims to analyze the effects of physical activity levels on shoulder pain and self-estimation improvement during the early postoperative period following ARCR and to investigate whether these effects differ between male and female patients. By comparing recovery patterns between sexes, this study seeks to elucidate the interaction between physical activity and postoperative recovery in a sex-specific manner. Ultimately, this research aims to enhance the understanding of sex-related differences in self-estimation improvement after ARCR and to explore the potential implications of incorporating sex-specific considerations into rehabilitation strategies to optimize patient outcomes. We hypothesize that individuals with higher physical activity levels will demonstrate lower shoulder pain and greater self-estimation in the early postoperative phase following ARCR and that these effects may differ between male and female patients. However, as a retrospective observational analysis, this study does not aim to establish direct causal relationships but rather to explore potential associations that may inform future prospective research.

## 2. Methods

### 2.1. Participants

From March 2019 to February 2021, a total of 318 patients underwent ARCR performed by the same orthopedic surgeon. To minimize pharmacological confounding in pain-related outcomes, patients who required opioid use beyond one week after surgery were excluded during the initial identification phase. In accordance with institutional postoperative pain management protocols, all patients were prescribed acetaminophen and nonsteroidal anti-inflammatory drugs (NSAIDs) as first-line analgesics. To account for pathological and psychosocial factors that could influence postoperative outcomes, the following exclusion criteria were applied: patients with massive or small rotator cuff tears, those with subscapularis tears, patients undergoing revision ARCR for retears, those who had undergone concomitant surgeries on the same shoulder, and patients with workers’ compensation claims. As a result, 125 patients were excluded, and a total of 193 patients were included in this retrospective study. All patients included in the study underwent ARCR using the suture bridge technique along with subacromial decompression. Subacromial decompression is a procedure that increases the height of the subacromial space to alleviate impingement on the rotator cuff tendons.

In this retrospective observational study, we sought to investigate the natural variation in physical activity levels among patients during the early postoperative period and their association with shoulder pain and functional outcomes following ARCR. All patients completed the International Physical Activity Questionnaire (IPAQ) weekly for six weeks postoperatively, providing a descriptive overview of activity levels without experimental manipulation. Given the ethical and practical limitations of intervening in physical activity during the critical early healing phase (4–6 weeks), our study was designed as an observational analysis focusing on naturally occurring patient behavior within this period. The IPAQ is a validated and reliable tool for assessing physical activity levels and consists of seven questions evaluating the frequency and duration of vigorous physical activity, moderate physical activity, walking activity, and inactivity over the past seven days [21]. Due to postoperative shoulder immobilization, only lower extremity activities were included in the analysis, such as walking, stair climbing, jogging, and stationary cycling. This approach was intended to reflect feasible and clinically relevant activity patterns during the early postoperative phase, while minimizing interference with tendon healing at the surgical site. The recorded physical activity data were converted into weekly MET-min values using the formula proposed by Ainsworth et al. [22]. The standard metabolic equivalent of task (MET) values used for calculation were 3.3 METs for walking, 4.0 METs for moderate physical activity, and 8.0 METs for vigorous physical activity. Moderate physical activity was defined as exercise that requires a moderate level of exertion and causes slight breathlessness (e.g., brisk walking, cycling at a moderate pace), whereas vigorous physical activity was defined as exercise that requires a high level of exertion and causes substantial breathlessness (e.g., fast stair climbing, fast cycling).

To classify patients into high and low physical activity groups, we calculated the mean weekly MET-min values based on IPAQ responses collected over this six-week immobilization phase. This approach was intended to account for temporal fluctuations and provide a more stable and representative indicator of actual activity levels during this constrained early postoperative period. Patients who achieved at least 600 MET-min per week through walking or moderate physical activity on five or more days or who performed vigorous physical activity for at least 20 min on three or more days per week were categorized into the high physical activity (HPA) group, while those who did not meet these criteria were assigned to the low physical activity (LPA) group. Each group was subsequently subdivided by sex to examine sex-based differences in physical activity levels and self-estimation.

Body composition parameters, including skeletal muscle mass, fat mass, body mass index (BMI), and body fat percentage, were assessed preoperatively using bioelectrical impedance analysis (BIA) with the InBody 720 device (Biospace Co., Ltd., Seoul, Republic of Korea). Measurements were conducted by trained personnel following the manufacturer’s standard protocol. All participants were instructed to refrain from food, drink, and strenuous exercise for at least 4 h prior to measurement. This method has been previously validated for estimating body composition in clinical and orthopedic populations [23].

All patients underwent assessments of shoulder pain, range of motion (ROM), and subjective self-estimation preoperatively and at 6 weeks, 3 months, and 6 months postoperatively. Isokinetic shoulder strength was not assessed at 6 weeks to avoid excessive tension on the repaired tendon but was evaluated preoperatively and at 3 and 6 months postoperatively. This study received informed consent from all participants and was approved by the Institutional Ethics Committee of Seoul Paik Hospital (PAIK 2021-04-009).

### 2.2. Subjective Shoulder Pain

Shoulder pain was assessed using the Visual Analog Scale (VAS), a reliable and widely used tool designed to allow patients to subjectively quantify their pain intensity. The VAS is particularly useful for visually representing changes in pain perception experienced during activities of daily living and exercise [24]. The VAS employs a 10 cm linear scale with decimal precision, where the leftmost end (0 points) represents “no pain,” and the rightmost end (10 points) represents “the most severe pain imaginable.” Patients were instructed to mark their perceived pain intensity on the scale, and the position of the mark was subsequently quantified and analyzed by the researchers.

### 2.3. Subjective Self-Estimation

Subjective self-estimation related to activities of daily living was assessed using the American Shoulder and Elbow Surgeons (ASESs) score. In this study, “self-estimation” refers to the patient’s subjective perception of their own functional recovery and ability to perform daily tasks, as measured by the ASES questionnaire. The ASES is a widely used and highly reliable tool that evaluates both subjective pain and functional status [25]. It consists of a Visual Analog Scale (VAS)-based pain assessment and a 10-item questionnaire evaluating daily functional abilities. The total score is calculated by assigning equal weight to pain (50%) and function (50%), with a maximum score of 100 points; higher scores indicate better self-estimation. The ASES score has been validated in numerous studies, demonstrating high reliability and validity. Due to its strong reproducibility and ease of clinical application, it is widely utilized for evaluating various shoulder conditions. Previous studies assessing its reliability and clinical utility have reported an intraclass correlation coefficient (ICC) ranging from 0.84 to 0.96 and a minimum clinically important difference (MCID) of 6.4 points [26].

### 2.4. Isokinetic Shoulder Strength

Isokinetic shoulder strength was assessed using the HUMAC NORM (CSMi Inc., MA, USA) to evaluate extension, flexion, internal rotation, and external rotation strength in both shoulders. While isokinetic testing requires patients to exert maximal effort, performing such assessments too early after surgery may compromise the healing of the repaired tendon or produce unreliable results due to pain-related muscle inhibition. Therefore, in the present study, the first isokinetic strength test was conducted at 12 weeks postoperatively, a time point when most patients have entered the proliferative-to-remodeling phase of tendon healing and experience significantly reduced pain levels. This approach aligns with previous studies suggesting that isokinetic evaluation is appropriate and safe after the early postoperative period, particularly beyond 10 to 12 weeks when pain has subsided to a level that does not significantly interfere with maximal voluntary contraction [1]. Additionally, Gulotta and Rodeo emphasized the importance of avoiding excessive mechanical stress on the repaired tendon during the early healing phase (up to 6–8 weeks), recommending delayed initiation of high-intensity assessments to ensure biological healing integrity [27]. In accordance with these recommendations, isokinetic testing was intentionally delayed until 12 weeks postoperatively in our protocol to ensure both the safety and reliability of measurements. Consequently, strength measurements were conducted at three time points: preoperatively, 12 weeks postoperatively, and 24 weeks postoperatively. Prior to the assessment, all participants received a detailed explanation and practical demonstrations of the testing procedure to ensure accurate execution. For the evaluation of shoulder flexion and extension strength, subjects were positioned supine on the testing apparatus, with the axis of the dynamometer aligned with the anatomical axis of the shoulder joint. The ROM was set from a neutral horizontal position (0°) to 120° of forward flexion. For the assessment of internal and external rotation strength, subjects were seated with their back supported by the testing device. The shoulder was positioned at 45° of abduction in the scapular plane, with the elbow flexed at 90°. The elbow was then secured to the dynamometer, and the ROM was set from 30° of internal rotation to 50° of external rotation, covering a total arc of 80°. Each test was performed at an angular velocity of 60°/sec, and a total of four repetitions were conducted to measure peak torque. The peak torque of the operated shoulder was normalized as a percentage relative to the contralateral, non-operated shoulder [28].

### 2.5. Postoperative Rehabilitation

A standardized postoperative rehabilitation protocol was applied to all patients in this study [29]. All rehabilitation sessions were conducted according to a standardized protocol, and although multiple physiotherapists were involved, they strictly adhered to this protocol to ensure consistency across patients. The rehabilitation process was uniformly initiated on postoperative day one for all participants. During the first six weeks postoperatively, patients wore an abduction brace to maintain the shoulder in 20° of abduction and 30° of internal rotation. During this period, only active ROM (AROM) exercises for adjacent joints and scapular positioning exercises, including scapular retraction and depression, were permitted. Beginning at six weeks postoperatively, patients initiated isometric shoulder exercises and passive ROM exercises, initially focusing on limited forward flexion and external rotation, with gradual progression in ROM. Between postoperative weeks 8 and 9, active-assisted ROM exercises were introduced, incorporating forward flexion and external rotation movements. From weeks 10 to 11, rehabilitation progressed to AROM exercises, including forward flexion, extension, external rotation, and internal rotation. Notably, to prevent excessive tensile load on the repaired tendon, all forms of internal rotation ROM exercises (both active and passive) were restricted until nine weeks postoperatively, and active internal rotation exercises commenced from week 10. Resistive strengthening exercises began at 12 weeks postoperatively when the repaired tendon was expected to have sufficient strength and tensile integrity. Initially, resistance exercises were performed using elastic bands, and the intensity was gradually increased by incorporating closed-chain exercises (Table 1).

### 2.6. Statistical Analysis

All collected data were analyzed using SPSS ver. 22.0 (IBM Corp., Armonk, NY, USA). Descriptive statistics, including mean and standard deviation (SD), were calculated. A two-way repeated measures analysis of variance (ANOVA) was performed to examine the main effects of time and group, as well as their interaction effects. For variables with significant interaction effects, post-hoc analyses were conducted. Multiple comparisons were performed to analyze intra-group differences over time and inter-group differences at each time point. Bonferroni correction was applied to adjust for Type I error inflation due to multiple comparisons. All statistical analyses were conducted to compare differences in physical activity levels between sexes, and the level of statistical significance was set at *p* < 0.05.

## 3. Results

### 3.1. General Characteristics of the Participants

**Figure 2:**
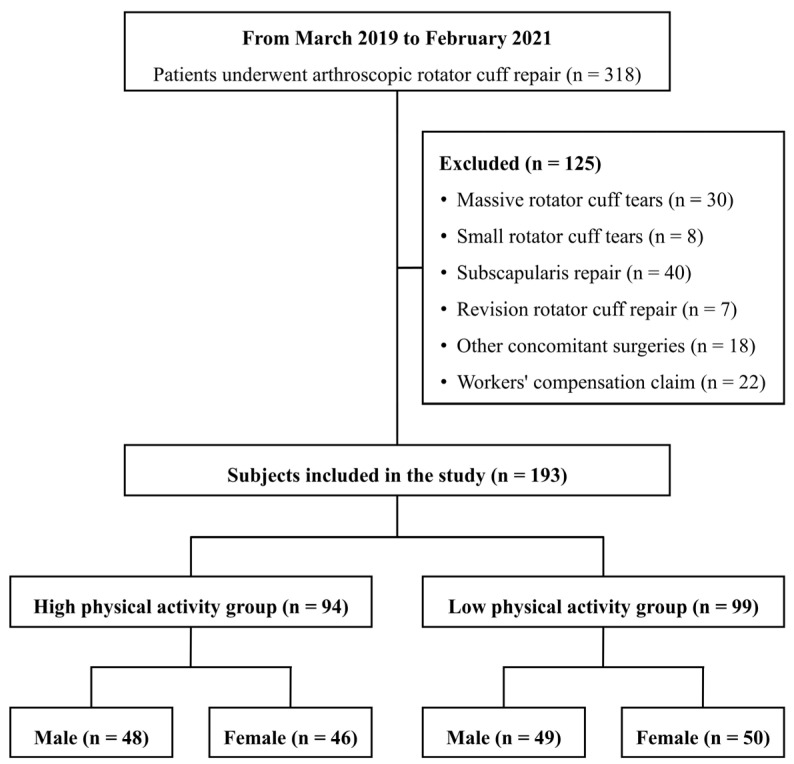
Diagram of patient flow.

Participants were classified into HPA (male = 48, female = 46) and LPA (male = 49, female = 50) groups based on their physical activity levels, and a comparative analysis of general characteristics between the two groups was conducted (Figure 2). Statistical analysis revealed no significant differences between HPA and LPA groups within each sex in terms of height, weight, skeletal muscle mass, fat mass, BMI, body fat percentage, involved side, or tear size. However, a significant difference was observed in weekly physical activity volume, which was the criterion for group classification (*p* < 0.05). The general characteristics of participants according to physical activity levels within each sex are presented in Table 2.

### 3.2. Subjective Shoulder Pain Based on Groups

The changes in subjective shoulder pain, assessed using the VAS, and inter-group comparisons over time following ARCR are presented in Table 3. Analysis revealed a statistically significant interaction effect between time and group (*p* < 0.05). Given the significant interaction, post-hoc analyses were performed. Within-group comparisons showed that all groups experienced a significant reduction in pain at 6, 12, and 24 weeks postoperatively compared to preoperative levels (*p* < 0.05). Inter-group comparisons at each time point confirmed that the HPA groups (both male and female) demonstrated significantly greater reductions in VAS scores compared to the LPA groups 6 weeks postoperatively (*p* < 0.05), indicating superior early pain relief in more physically active individuals. No significant differences were noted between sexes. At 12 weeks, the male LPA group achieved pain levels comparable to those of the HPA groups, while the female LPA group continued to report significantly higher pain levels (*p* < 0.05), suggesting delayed pain recovery in less active females. By 24 weeks postoperatively, no significant differences were observed among the four groups.

### 3.3. Subjective Self-Estimation Based on Groups

Changes in subjective self-estimation, as assessed by the ASES score, and inter-group comparisons over time following ARCR are presented in Table 4. Statistical analysis revealed a significant interaction effect between time and group (*p* < 0.05). Given the significant interaction, post-hoc analyses were performed. Within-group comparisons showed that all groups demonstrated a significant improvement in ASES scores at 12 and 24 weeks postoperatively compared to preoperative levels (*p* < 0.05). Inter-group comparisons at each time point indicated that at 6 and 12 weeks postoperatively, both male and female HPA groups exhibited significantly higher ASES scores than their respective LPA counterparts (*p* < 0.05), reflecting superior early self-estimation improvement in the more physically active groups. However, no significant sex-based differences were observed within each group. By 24 weeks postoperatively, self-estimation improvement had progressed similarly across all groups, and no significant differences were found among the four groups.

### 3.4. Isokinetic Shoulder Strength Based on Groups

Changes in isokinetic shoulder strength over time and inter-group differences following ARCR are presented in Table 5. The analysis revealed a statistically significant interaction effect between time and group for flexion and external rotation strength (*p* < 0.05), whereas no significant interaction was observed for extension and internal rotation strength. Within-group comparisons showed that all four groups experienced significant improvements in extension, flexion, internal rotation, and external rotation strength at 12 and 24 weeks postoperatively compared to preoperative values (*p* < 0.05). Inter-group comparisons at each time point demonstrated that at 12 weeks postoperatively, both male and female HPA groups exhibited significantly greater flexion and external rotation strength than their respective LPA groups (*p* < 0.05), suggesting superior early strength recovery in physically active individuals. By 24 weeks postoperatively, no significant differences were observed among the groups, indicating that the advantage observed in the HPA group during the early recovery phase gradually diminished over time.

## 4. Discussion

Arthroscopic rotator cuff repair (ARCR) is widely performed as an effective surgical intervention to restore self-estimation and alleviate pain. However, persistent challenges remain in the early postoperative phase, as patients often experience severe pain and significant functional decline, making early return to daily activities difficult [1]. Consequently, identifying safe and effective strategies to optimize pain management and functional recovery during the early healing phase is of critical importance. Recent studies suggest that physical activity may play a key role in these recovery processes [10,11]. Physical activity has been shown to not only reduce localized pain but also induce a generalized hypoalgesic effect, making it a promising adjunct to postoperative pain management [30]. Furthermore, some studies have suggested that the impact of physical activity on functional recovery may differ between males and females [19]. However, these findings remain largely inconclusive, and sex differences in postoperative outcomes are not yet fully understood. In the present study, we considered sex not merely as a potential confounding factor but as an a priori variable of interest, aiming to elucidate its potential moderating role in the relationship between physical activity and early postoperative recovery. This rationale guided our observational analysis, which was designed to explore potential associations rather than to establish direct causal relationships. Therefore, this study aimed to analyze the effects of physical activity level and sex on shoulder pain and functional recovery during the early recovery phase after ARCR, with the ultimate goal of informing more tailored and effective rehabilitation strategies.

In this study, all groups demonstrated a significant reduction in pain over time at 6, 12, and 24 weeks postoperatively. However, inter-group comparisons at each time point revealed that at 6 weeks, the HPA group exhibited significantly lower pain scores than the LPA group. Notably, at 12 weeks, male patients in the LPA group showed pain levels comparable to those in the HPA group, whereas female patients in the LPA group continued to report significantly higher pain levels. These results raise the possibility that the analgesic effects of physical activity may vary by sex. One possible hypothesis involves sex-based differences in pain sensitivity. According to Fillingim et al., females exhibit higher pain sensitivity than males due to differences in pain inhibitory brain regions and hormonal variations [31]. Similarly, Sodhi et al. reported that female patients experience greater perceived pain following total knee arthroplasty compared to males, which may be attributed to differences in neural processing and central nervous system responsiveness [32]. Such mechanisms may contribute to the observed trend that female patients in the LPA group experienced more prolonged pain, even under the same rehabilitation protocol. This observation aligns with previous evidence suggesting that women may require higher levels or more targeted forms of physical activity to achieve comparable analgesic effects. Tashjian et al. established the minimal clinically important difference (MCID) for VAS pain scores in rotator cuff disease as 1.5 points (*p* = 0.026) and the patient acceptable symptom state (PASS) as 3 points (95% CI, 2.27–3.73) [33]. In this study, by 24 weeks postoperatively, all groups demonstrated pain scores within the PASS threshold, and no significant inter-group differences were observed. However, at 6 weeks, patients in the HPA group exhibited more pronounced pain reduction, potentially reflecting early exercise-induced hypoalgesia. Ellingson et al. reported that maintaining recommended levels of physical activity is associated with decreased pain sensitivity and the modulation of central pain processing mechanisms [34]. Sluka et al. demonstrated that regular physical activity reduces neuronal excitability via the phosphorylation of NMDA receptors, modulates neuroimmune signaling, and enhances endogenous opioid and serotonin release in the brainstem pain inhibitory pathway [35]. Additionally, Koltyn et al. found that endogenous opioids, such as beta-endorphins, increase in peripheral circulation following physical activity and activate spinal cord inhibitory mechanisms to modulate pain [36]. Taken together, these mechanisms support the hypothesis that physical activity may influence pain regulation during the early postoperative period. The sustained higher pain levels among female patients in the LPA group highlight the potential importance of sex-specific considerations in designing rehabilitation strategies. Given these findings, early postoperative interventions should consider both physical activity levels and sex differences to enhance recovery outcomes.

This study evaluated the differences in subjective self-estimation recovery after ARCR by assessing ASES scores. The results indicated that all groups exhibited a gradual improvement in ASES scores over time, reflecting progressive recovery of self-estimation. Notably, both male and female patients in the HPA group demonstrated significantly higher ASES scores at 6 and 12 weeks postoperatively compared to the LPA group, suggesting that physical activity may contribute to enhanced functional recovery. Conversely, the LPA group showed a tendency for ASES scores to decrease at 6 weeks postoperatively compared to preoperative levels, implying that lower levels of physical activity may be associated with slower early functional recovery. These findings align with previous studies suggesting that physical activity plays a critical role in the physiological mechanisms underlying early functional recovery [37]. A recent study by Lee and Lim et al. analyzed the impact of early physical activity on functional recovery and pain management following hip fracture surgery in older adults. Their findings demonstrated that high-intensity rehabilitation programs were effective in preventing muscle atrophy and ultimately promoting functional recovery [37]. In this study, there were no statistically significant differences in ASES scores between males and females within the HPA and LPA groups at 6 and 12 weeks postoperatively, indicating that physical activity level, rather than sex, may be a more influential factor in functional recovery. Previous research has reported that females tend to have higher pain sensitivity and lower muscle mass than males, which may contribute to slower functional recovery [31]. However, in this study, no significant differences were observed between males and females, suggesting that maintaining an adequate level of physical activity may minimize potential sex-related disparities in functional recovery. At 24 weeks postoperatively, no significant differences in ASES scores were observed between the HPA and LPA groups or between sexes, indicating that functional recovery was achieved across all groups over time. Nevertheless, given that the HPA group exhibited superior functional recovery during the early postoperative phase, the influence of physical activity on early recovery speed may have important clinical implications. These findings suggest that postoperative physical activity levels may play a crucial role in functional recovery, particularly during the early rehabilitation phase. Moreover, the results indicate that sex-related differences in functional recovery may not be prominent when an appropriate level of physical activity is maintained. Thus, maintaining physical activity during early postoperative rehabilitation may be essential for optimizing recovery outcomes, regardless of sex.

This study analyzed the changes in isokinetic shoulder strength recovery after ARCR according to physical activity levels and sex. The results indicated that flexion, extension, internal rotation, and external rotation strength gradually increased over time across all groups. Notably, at 12 weeks postoperatively, the HPA group demonstrated significantly greater flexion and external rotation strength than the LPA group, whereas this difference was no longer significant at 24 weeks, suggesting a convergence over time. In contrast, no significant differences were observed in extension and internal rotation strength based on physical activity levels. The absence of significant differences in extension and internal rotation strength may be interpreted in the context of the functional roles of different shoulder muscles. The rotator cuff complex helps to keep the humeral head centered in the glenoid socket and provides dynamic shoulder stability during movement [38]. Wattanaprakornkul et al. conducted an electromyography study and found that, similar to the posterior and anterior rotator cuff muscles contributing to external and internal rotation, respectively, they also play a stabilizing role during flexion and extension movements [39]. In this study, patients with subscapularis tears were excluded, and all participants underwent ARCR for supraspinatus tears. Therefore, flexion and external rotation strength, which are functions likely affected by the condition of the supraspinatus, were likely significantly weakened preoperatively. Consequently, these strength measures may have been more responsive to postoperative physical activity than extension and internal rotation strength. By 24 weeks postoperatively, no significant differences in strength were observed between groups, suggesting that even patients with lower physical activity levels eventually regained strength over time. This finding implies that physical activity levels may not be the primary determinant of long-term strength recovery. This aligns with the findings of Lee et al., who reported that differences in early rehabilitation strategies had a diminishing impact on functional recovery six months after rotator cuff repair [40]. The findings of this study suggest that physical activity levels may influence early strength recovery following rotator cuff repair, particularly during the first 12 weeks of rehabilitation. Maintaining physical activity during this period may play a crucial role in facilitating strength restoration.

The results of this study provide valuable insights into the role of physical activity in early functional recovery, emphasizing the importance of maintaining physical activity during initial rehabilitation. However, several limitations must be considered when interpreting and applying these findings. First, as a retrospective observational study, physical activity levels were not experimentally controlled or intentionally manipulated but were based on naturally occurring patient behaviors, reported weekly through a validated questionnaire (IPAQ). Consequently, the study had limitations in fully accounting for factors that may influence physical activity levels, such as underlying medical conditions, occupational demands, socioeconomic background, and individual motivation, which were not measured or adjusted for in the analysis. In particular, the inability to perform subgroup analyses based on age or overall health status, such as comorbidity burden or pre-existing physical capacity, may have introduced additional variability into the observed associations. Moreover, we treated sex-based groupings as a key variable of interest based on known differences in pain perception, physical activity patterns, and recovery trajectories. However, because we did not adjust for other potential confounders, these results should be interpreted as preliminary associations rather than definitive causal conclusions. Furthermore, because pain intensity can directly influence physical activity, the lack of systematic adjustment for individual pain levels may have confounded the observed associations between physical activity and functional recovery. Nevertheless, to enhance the reliability of the study, internationally validated tools such as the IPAQ were used to assess physical activity levels on a weekly basis during the early postoperative phase.

Additionally, the study was conducted at a single institution with a patient population limited to those undergoing surgery at a specific hospital, potentially limiting the generalizability of the findings. However, the study ensured internal validity by recruiting patients who underwent the same surgical procedure and received standardized postoperative care and rehabilitation protocols. Future studies incorporating multi-center research and larger, more diverse populations will be necessary to enhance the external validity of these findings. Moreover, while this study analyzed sex-related differences in functional recovery, it did not separately measure physiological factors such as muscle mass or hormonal changes, which could influence recovery trajectories between males and females. Despite this limitation, this study contributes to the understanding of the relationship between physical activity levels and functional recovery by comparing these effects across sexes. Importantly, future prospective, multi-center studies incorporating multivariate analyses and adjusting for broader background variables will be essential to more definitively isolate the independent effects of lower limb physical activity on early postoperative outcomes. Additionally, incorporating physiological and metabolic measures may provide a more comprehensive analysis and a deeper understanding of sex-based variations in functional recovery.

## 5. Conclusions

This study analyzed differences in self-estimation improvement following ARCR based on physical activity levels and sex. The results demonstrated that patients with higher physical activity levels experienced significantly lower pain and better self-estimation at 6 and 12 weeks postoperatively compared to those with lower physical activity levels. Notably, a significant difference in subjective shoulder pain was observed according to physical activity levels. At 12 weeks postoperatively, the male LPA group showed pain levels comparable to those of the HPA group, whereas the female LPA group continued to report significantly higher pain. These findings suggest a possible association between physical activity and early pain modulation, particularly in the context of sex-related differences. While causal relationships cannot be established due to the observational nature of the study, the results imply that female patients may benefit from more active engagement in postoperative rehabilitation. Overall, the findings support the potential relevance of maintaining physical activity during the initial recovery phase and underscore the importance of considering sex-specific patterns in postoperative pain experience.

## Figures and Tables

**Table 1:** Postoperative rehabilitation program.

**Period**	**HPA**	**LPA**
0–5 weeks	Initiation of AROM exercises for adjacent joints (cervical spine, elbow, wrist, and hand)	
	Emphasis on scapular stabilization (retraction and depression)	
6–7 weeks	Initiation of PROM exercises for the shoulder (FF, ER)	
	Emphasis on shoulder isometric strengthening exercises (FF, extension, ER, IR)	
8–9 weeks	Transition to AAROM exercises for the shoulder (FF, ER)	
	Table slides (FF, Scaption)	
10–11 weeks	Progression to AROM for the shoulder (FF, extension, ER, IR)	
	Progression from supported vertical wall slides to wall walks	
12–24 weeks	Advancement to pain-free full PROM, followed by a transition to full AROM	
	Integration of progressive resistance training (elastic resistance band exercise, etc.)	

HPA, high physical activity group; LPA, low physical activity group; AROM, active range of motion; PROM, passive range of motion; FF, forward flexion; ER, external rotation; IR, internal rotation; AAROM, active assisted range of motion.

**Table 2:** General characteristics of participants.

Variables	HPA (n = 94)		*p*	LPA (n = 99)		*p*
	Male (n = 48)	Female (n = 46)		Male (n = 49)	Female (n = 50)	
Age (year)	59.0 ± 9.3	60.3 ± 9.7	0.610	60.1 ± 7.6	61.1 ± 2.5	0.361
Height (cm)	169.8 ± 6.1	170.1 ± 3.0	0.743	154.3 ± 4.7	153.5 ± 3.6	0.511
Weight (kg)	72.9 ± 3.8	71.9 ± 4.0	0.267	57.8 ± 8.2	59.6 ± 5.7	0.851
Skeletal muscle mass (kg)	29.7 ± 3.0	30.0 ± 2.1	0.880	20.7 ± 3.0	21.2 ± 2.9	0.733
Fat mass (kg)	24.6 ± 5.1	24.7 ± 2.9	0.546	19.9 ± 5.0	20.7 ± 3.4	0.619
BMI (kg/m^2^)	25.3 ± 1.3	24.6 ± 1.5	0.138	24.6 ± 3.1	25.2 ± 2.1	0.619
Body fat percentage (%)	33.1 ± 6.0	33.5 ± 3.9	0.240	33.2 ± 6.9	35.3 ± 5.3	0.409
Involved side, n (%)						
Right	26 (54.2)	24 (52.2)	0.980	25 (51.0)	27 (54.0)	0.919
Left	22 (45.8)	22 (47.8)		24 (49.0)	23 (46.0)	
Tear size, n (%)						
Medium	26 (54.2)	28 (60.9)	0.479	29 (59.2)	27 (54.0)	0.560
Large	22 (45.8)	18 (39.1)		20 (40.8)	23 (46.0)	
PA volume (MET·min/wk)	1029.1 ± 231.3	401.1 ± 65.3	<0.001 *	1089.9 ± 258.1	379.6 ± 99.9	<0.001 *

* *p* < 0.05; HPA, high physical activity group; LPA, low physical activity group; BMI, body mass index; PA, physical activity.

**Table 3:** Sex differences in subjective shoulder pain based on physical activity level.

Variables	Group	Preop	6 wk	12 wk	24 wk	*p*
VAS, score	MHPA	7.53 ± 0.70	3.86 ± 0.57 ^a,d,e†,‡^	2.75 ± 0.80 ^b,d,f‡^	1.30 ± 0.84 ^c,e,f^	T: <0.001 * G: <0.001 * T×G: <0.001 *
	FHPA	8.07 ± 0.54	3.97 ± 0.79 ^a,d,e§,#^	3.07 ± 1.07 ^b,d,f#^	1.27 ± 1.03 ^c,e,f^	
	MLPA	7.41 ± 0.85	6.08 ± 1.02 ^a,d,e†,§^	4.19 ± 1.53 ^b,d,f^	1.75 ± 1.17 ^c,e,f^	
	FLPA	7.97 ± 0.79	6.87 ± 0.71 ^a,d,e‡,#^	4.77 ± 1.66 ^b,d,f‡,#^	2.37 ± 1.68 ^c,e,f^	

* *p* < 0.05; VAS, visual analog scale; MHPA, male high physical activity group; FHPA, female high physical activity group; MLPA, male low physical activity group; FLPA, female low physical activity group; Preop: preoperation, wk: weeks; T, time; G, group; _a_ Preop vs. 6wk, _b_ Preop vs. 12wk, _c_ Preop vs. 24wk, _d_ 6wk vs. 12wk, _e_ 6wk vs. 24wk; _f_ 12wk vs. 24wk; ^†^ MHPA vs. MLPA; ^‡^ MHPA vs. FLPA; ^§^ FHPA vs. MLPA; ^#^ FHPA vs. FLPA.

**Table 4:** Sex differences in subjective self-estimation based on physical activity level.

Variables	Group	Preop	6 wk	12 wk	24 wk	*p*
ASES, score	MHPA	39.2 ± 3.6	44.5 ± 6.4 ^d,e†,‡^	64.4 ± 3.5 ^b,d,f†,‡^	77.8 ± 8.3 ^c,e,f^	T: <0.001 * G: 0.005 * T×G: 0.029 *
	FHPA	38.2 ± 9.8	43.4 ± 2.5 ^d,e§,#^	65.4 ± 8 ^b,d,f§,#^	78.6 ± 9.7 ^c,e,f^	
	MLPA	38.9 ± 6.6	34.1 ± 9.8 ^d,e†,§^	54.7 ± 1.8 ^b,d,f†,§^	75 ± 5.2 ^c,e,f^	
	FLPA	38.6 ± 6.7	35.2 ± 3.2 ^d,e‡,#^	53 ± 7.6 ^b,d,f‡,#^	74.5 ± 7.0 ^c,e.f^	

*p < 0.05; ASES, American shoulder and elbow surgeons score; MHPA, male high physical activity group; FHPA, female high physical activity group; MLPA, male low physical activity group; FLPA, female low physical activity group; Preop: preoperation, wk: weeks; T, time; G, group; _b_ Preop vs. 12wk, _c_ Preop vs. 24wk, _d_ 6wk vs. 12wk, _e_ 6wk vs. 24wk; _f_ 12wk vs. 24wk; ^†^ MHPA vs. MLPA; ^‡^ MHPA vs. FLPA; ^§^ FHPA vs. MLPA; ^#^ FHPA vs. FLPA.

**Table 5:** Sex differences in isokinetic shoulder strength based on physical activity level.

Variables	Group	Preop	12 wk	24 wk	*p*
Extension, %	MHPA	77.1 ± 2.5	82.5 ± 1.7 ^a,c^	96.1 ± 0.9 ^b,c^	T: <0.001 * G: 0.396 T×G: 0.754
	FHPA	76.8 ± 4.7	81.7 ± 2.0 ^a,c^	94.9 ± 2.4 ^b,c^	
	MLPA	77.0 ± 2.1	80.5 ± 5.7 ^a,c^	95.1 ± 1.6 ^b,c^	
	FLPA	77.2 ± 2.1	79.8 ± 2.0 ^a,c^	94.6 ± 1.5 ^b,c^	
Flexion, %	MHPA	59.0 ± 4.6	71.0 ± 3.0 ^a,c†,‡^	78.0 ± 1.9 ^b,c^	T: <0.001 * G: 0.006 * T×G: <0.001 *
	FHPA	58.6 ± 3.2	69.9 ± 2.4 ^a,c§,#^	77.7 ± 2.6 ^b,c^	
	MLPA	58.2 ± 4.3	63.6 ± 2.2 ^a,c†,§^	76.8 ± 2.4 ^b,c^	
	FLPA	58.8 ± 4.3	63.3 ± 2.6 ^a,c‡,#^	75.9 ± 2.6 ^b,c^	
Internal rotation, %	MHPA	71.0 ± 3.5	78.6 ± 3.8 ^a,c^	87.0 ± 2.7 ^b,c^	T: <0.001 * G: 0.486 T×G: 0.799
	FHPA	70.3 ± 4.6	78.1 ± 3.6 ^a,c^	85.9 ± 1.3 ^b,c^	
	MLPA	71.9 ± 1.8	77.6 ± 2.7 ^a,c^	86.5 ± 2.1 ^b,c^	
	FLPA	70.9 ± 3.0	76.8 ± 2.5 ^a,c^	85.4 ± 1.1 ^b,c^	
External rotation, %	MHPA	50.1 ± 3.2	68.1 ± 2.2 ^a,c†,‡^	78.8 ± 3.5 ^b,c^	T: <0.001 * G: 0.059 T×G: <0.001 *
	FHPA	49.3 ± 2.9	67.7 ± 3.4 ^a,c§,#^	79.1 ± 3.9 ^b,c^	
	MLPA	51.1 ± 2.4	60.6 ± 5.7 ^a,c†,§^	78.6 ± 4.2 ^b,c^	
	FLPA	49.1 ± 4.2	59.9 ± 3.5 ^a,c‡,#^	78.0 ± 5.1 ^b,c^	

* *p* < 0.05; MHPA, male high physical activity group; FHPA, female high physical activity group; MLPA, male low physical activity group; FLPA, female low physical activity group; Preop: preoperation, wk: weeks; T, time; G, group; _a_ Preop vs. 6wk, _b_ Preop vs. 12wk, _c_ Preop vs. 24wk; ^†^ MHPA vs. MLPA; ^‡^ MHPA vs. FLPA; ^§^ FHPA vs. MLPA; ^#^ FHPA vs. FLPA.

## Data Availability

The data presented in this study are available on reasonable request from the corresponding author.

## References

[1] Nikolaidou O., Migkou S., Karampalis C. (2017). Suppl-1, M9: Rehabilitation after rotator cuff repair. Open Orthop. J..

[2] Kuhn J.E. (2023). Prevalence, natural history, and nonoperative treatment of rotator cuff disease. Oper. Tech. Sports Med..

[3] Moran T.E., Werner B.C. (2023). Surgery and rotator cuff disease: A review of the natural history, indications, and outcomes of nonoperative and operative treatment of rotator cuff tears. Clin. Sports Med..

[4] Dey Hazra R.-O., Ernat J.J., Rakowski D.R., Boykin R.E., Millett P.J. (2021). The evolution of arthroscopic rotator cuff repair. Orthop. J. Sports Med..

[5] Uquillas C.A., Capogna B.M., Rossy W.H., Mahure S.A., Rokito A.S. (2016). Postoperative pain control after arthroscopic rotator cuff repair. J. Shoulder Elb. Surg..

[6] Mandava N.K., Sethi P.M., Routman H.D., Liddy N., Haidamous G., Denard P.J. (2021). Opioid requirement after rotator cuff repair is low with a multimodal approach to pain. J. Shoulder Elb. Surg..

[7] Davis W.H., Sandler A.B., Scanaliato J.P., Dunn J.C., Parnes N. (2022). Use of opioids in the early postoperative period after arthroscopic rotator cuff repair: A systematic review. Orthop. J. Sports Med..

[8] Connizzo B.K., Yannascoli S.M., Tucker J.J., Caro A.C., Riggin C.N., Mauck R.L., Soslowsky L.J., Steinberg D.R., Bernstein J. (2014). The detrimental effects of systemic Ibuprofen delivery on tendon healing are time-dependent. Clin. Orthop. Relat. Res..

[9] Yung E., Got T., Patel N., Brull R., Abdallah F. (2021). Intra-articular infiltration analgesia for arthroscopic shoulder surgery: A systematic review and meta-analysis. Anaesthesia.

[10] Tan L., Cicuttini F.M., Fairley J., Romero L., Estee M., Hussain S.M., Urquhart D.M. (2022). Does aerobic exercise effect pain sensitisation in individuals with musculoskeletal pain? A systematic review. BMC Musculoskelet. Disord..

[11] Song J.S., Yamada Y., Kataoka R., Wong V., Spitz R.W., Bell Z.W., Loenneke J.P. (2022). Training-induced hypoalgesia and its potential underlying mechanisms. Neurosci. Biobehav. Rev..

[12] Tomschi F., Ransmann P., Schmidt A., Hilberg T. (2024). Exercise induced hypoalgesia after a high intensity functional training: A randomized controlled crossover study. BMC Sports Sci. Med. Rehabil..

[13] Naugle K.M., Naugle K.E., Teegardin M., Kaleth A.S. (2023). Physical activity to prevent the age-related decline of endogenous pain modulation. Exerc. Sport Sci. Rev..

[14] Leung A., Gregory N.S., Allen L.-A.H., Sluka K.A. (2016). Regular physical activity prevents chronic pain by altering resident muscle macrophage phenotype and increasing interleukin-10 in mice. Pain.

[15] Littlewood C., Bateman M. (2015). Rehabilitation following rotator cuff repair: A survey of current UK practice. Shoulder Elb..

[16] Parsons B.O., Gruson K.I., Chen D.D., Harrison A.K., Gladstone J., Flatow E.L. (2010). Does slower rehabilitation after arthroscopic rotator cuff repair lead to long-term stiffness?. J. Shoulder Elb. Surg..

[17] Sahoo S., Ricchetti E.T., Zajichek A., Group C.C.S., Evans P.J., Farrow L.D., McCoy B.W., Jones M.H., Miniaci A.A., Sabesan V.J. (2020). Associations of preoperative patient mental health and sociodemographic and clinical characteristics with baseline pain, function, and satisfaction in patients undergoing rotator cuff repairs. Am. J. Sports Med..

[18] Saccomanno M.F., Sircana G., Cazzato G., Donati F., Randelli P., Milano G. (2016). Prognostic factors influencing the outcome of rotator cuff repair: A systematic review. Knee Surg. Sports Traumatol. Arthrosc..

[19] Daniels S.D., Stewart C.M., Garvey K.D., Brook E.M., Higgins L.D., Matzkin E.G. (2019). Sex-based differences in patient-reported outcomes after arthroscopic rotator cuff repair. Orthop. J. Sports Med..

[20] Rice D., Nijs J., Kosek E., Wideman T., Hasenbring M.I., Koltyn K., Graven-Nielsen T., Polli A. (2019). Exercise-induced hypoalgesia in pain-free and chronic pain populations: State of the art and future directions. J. Pain.

[21] Sember V., Meh K., Sorić M., Starc G., Rocha P., Jurak G. (2020). Validity and reliability of international physical activity questionnaires for adults across EU countries: Systematic review and meta analysis. Int. J. Environ. Res. Public Health.

[22] Ainsworth B.E., Haskell W.L., Herrmann S.D., Meckes N., Bassett D.R., Tudor-Locke C., Greer J.L., Vezina J., Whitt-Glover M.C., Leon A.S. (2011). 2011 Compendium of Physical Activities: A second update of codes and MET values. Med. Sci. Sports Exerc..

[23] Looney D.P., Schafer E.A., Chapman C.L., Pryor R.R., Potter A.W., Roberts B.M., Friedl K.E. (2024). Reliability, biological variability, and accuracy of multi-frequency bioelectrical impedance analysis for measuring body composition components. Front. Nutr..

[24] Tashjian R.Z., Shin J., Broschinsky K., Yeh C.-C., Martin B., Chalmers P.N., Greis P.E., Burks R.T., Zhang Y. (2020). Minimal clinically important differences in the American Shoulder and Elbow Surgeons, Simple Shoulder Test, and visual analog scale pain scores after arthroscopic rotator cuff repair. J. Shoulder Elb. Surg..

[25] Cunningham G., Lädermann A., Denard P.J., Kherad O., Burkhart S.S. (2015). Correlation between American shoulder and elbow surgeons and single assessment numerical evaluation score after rotator cuff or SLAP repair. Arthrosc. J. Arthrosc. Relat. Surg..

[26] Wylie J.D., Beckmann J.T., Granger E., Tashjian R.Z. (2014). Functional outcomes assessment in shoulder surgery. World J. Orthop..

[27] Gulotta L.V., Rodeo S.A. (2009). Growth factors for rotator cuff repair. Clin. Sports Med..

[28] Bigoni M., Gorla M., Guerrasio S., Brignoli A., Cossio A., Grillo P., Marinoni E.C. (2009). Shoulder evaluation with isokinetic strength testing after arthroscopic rotator cuff repairs. J. Shoulder Elb. Surg..

[29] Sgroi T.A., Cilenti M. (2018). Rotator cuff repair: Post-operative rehabilitation concepts. Curr. Rev. Musculoskelet. Med..

[30] Naugle K.M., Fillingim R.B., Riley J.L. (2012). A meta-analytic review of the hypoalgesic effects of exercise. J. Pain.

[31] Fillingim R.B., King C.D., Ribeiro-Dasilva M.C., Rahim-Williams B., Riley J.L. (2009). Sex, gender, and pain: A review of recent clinical and experimental findings. J. Pain.

[32] Sodhi N., Qilleri A., Aprigliano C., Danoff J.R. (2024). One Size Does Not Fit All: Women Experience More Pain Than Men after Total Knee Arthroplasty. J. Arthroplast..

[33] Tashjian R.Z., Deloach J., Porucznik C.A., Powell A.P. (2009). Minimal clinically important differences (MCID) and patient acceptable symptomatic state (PASS) for visual analog scales (VAS) measuring pain in patients treated for rotator cuff disease. J. Shoulder Elb. Surg..

[34] Ellingson L.D., Colbert L.H., Cook D.B. (2012). Physical activity is related to pain sensitivity in healthy women. Med. Sci. Sports Exerc..

[35] Sluka K.A., O’Donnell J.M., Danielson J., Rasmussen L.A. (2013). Regular physical activity prevents development of chronic pain and activation of central neurons. J. Appl. Physiol..

[36] Koltyn K.F., Brellenthin A.G., Cook D.B., Sehgal N., Hillard C. (2014). Mechanisms of exercise-induced hypoalgesia. J. Pain.

[37] Lee N., Lim W. (2025). Effects of a subacute high-intensity rehabilitation program in older adult inpatients following intramedullary nailing for hip fractures. J. Bodyw. Mov. Ther..

[38] Vidt M.E., Santago A.C., Marsh A.P., Hegedus E.J., Tuohy C.J., Poehling G.G., Freehill M.T., Miller M.E., Saul K.R. (2018). Modeling a rotator cuff tear: Individualized shoulder muscle forces influence glenohumeral joint contact force predictions. Clin. Biomech..

[39] Wattanaprakornkul D., Cathers I., Halaki M., Ginn K.A. (2011). The rotator cuff muscles have a direction specific recruitment pattern during shoulder flexion and extension exercises. J. Sci. Med. Sport.

[40] Lee B.G., Cho N.S., Rhee Y.G. (2012). Effect of two rehabilitation protocols on range of motion and healing rates after arthroscopic rotator cuff repair: Aggressive versus limited early passive exercises. Arthrosc. J. Arthrosc. Relat. Surg..

